# Customized Tracking Algorithm for Robust Cattle Detection and Tracking in Occlusion Environments

**DOI:** 10.3390/s24041181

**Published:** 2024-02-11

**Authors:** Wai Hnin Eaindrar Mg, Pyke Tin, Masaru Aikawa, Ikuo Kobayashi, Yoichiro Horii, Kazuyuki Honkawa, Thi Thi Zin

**Affiliations:** 1 Interdisciplinary Graduate School of Agriculture and Engineering, University of Miyazaki, Miyazaki 889-2192, Japan; nc22003@student.miyazaki-u.ac.jp; 2 Graduate School of Engineering, University of Miyazaki, Miyazaki 889-2192, Japan; pyketin11@gmail.com; 3 Organization for Learning and Student Development, University of Miyazaki, Miyazaki 889-2192, Japan; 4 Sumiyoshi Livestock Science Station, Faculty of Agriculture, University of Miyazaki, Miyazaki 889-2192, Japan; 5 Center for Animal Disease Control, University of Miyazaki, Miyazaki 889-2192, Japan; horii@cc.miyazaki-u.ac.jp; 6 Honkawa Ranch, Oita 877-0056, Japan

**Keywords:** cattle detection, tracking, customized tracking algorithm (CTA), occlusion, track-ID increment cases, miss detection

## Abstract

Ensuring precise calving time prediction necessitates the adoption of an automatic and precisely accurate cattle tracking system. Nowadays, cattle tracking can be challenging due to the complexity of their environment and the potential for missed or false detections. Most existing deep-learning tracking algorithms face challenges when dealing with track-ID switch cases caused by cattle occlusion. To address these concerns, the proposed research endeavors to create an automatic cattle detection and tracking system by leveraging the remarkable capabilities of Detectron2 while embedding tailored modifications to make it even more effective and efficient for a variety of applications. Additionally, the study conducts a comprehensive comparison of eight distinct deep-learning tracking algorithms, with the objective of identifying the most optimal algorithm for achieving precise and efficient individual cattle tracking. This research focuses on tackling occlusion conditions and track-ID increment cases for miss detection. Through a comparison of various tracking algorithms, we discovered that Detectron2, coupled with our customized tracking algorithm (CTA), achieves 99% in detecting and tracking individual cows for handling occlusion challenges. Our algorithm stands out by successfully overcoming the challenges of miss detection and occlusion problems, making it highly reliable even during extended periods in a crowded calving pen.

## 1. Introduction

Cattle detection and tracking play a crucial role in various domains, such as precision agriculture, livestock management, and animal behavior analysis. The ability to accurately detect and track individual cows in real time provides valuable insights for monitoring health and behavior patterns and can be used to optimize farming practices [1,2]. The complex nature of the cattle environment, coupled with the potential for missed or false detections, poses significant obstacles to existing deep-learning tracking algorithms. One challenge that arises in cattle tracking is the occurrence of occlusion [3,4,5], where cattle may temporarily obstruct each other’s visibility. This occlusion can lead to track-ID switch cases, where the tracking algorithm mistakenly assigns different IDs to the same cow or fails to associate the correct ID when a cow becomes visible again [6]. Such track-ID switch cases can disrupt the continuity of tracking and cause inaccuracies in the tracking results.

To address these concerns, this research focuses on developing an automatic cattle detection and tracking system that effectively handles occlusion conditions and track-ID increment cases [7]. By customizing and fine-tuning the Detectron2 model with cow-specific datasets, we aim to enhance the algorithm’s performance in accurately locating and identifying cows, even in challenging environments [8]. Furthermore, this study conducts an extensive comparison of eight distinct deep-learning tracking algorithms to identify the most optimal approach for precise and efficient individual cattle tracking. Subsequently, we introduce a novel monitoring method tailored for robust cattle detection and tracking in occlusion environments. Our method integrates advanced computer vision techniques, including Detectron2, along with a suite of eight different algorithms, including the CTA. These techniques are specifically designed to detect and track cattle in challenging conditions characterized by frequent occlusions. Our approach innovatively combines Detectron2 with various algorithms, notably CTA, to significantly improve the reliability and accuracy of cattle monitoring in real-world scenarios characterized by occlusions. The key contributions of our study can be summarized as follows:

(1) Development of a general framework for cattle detection and segmentation: the study proposes a framework that utilizes tailored modifications embedded in Detectron2, leveraging scientific principles such as mask region-based convolutional neural networks (Mask R-CNNs) to enhance the accuracy and efficiency of cattle detection and segmentation in diverse agricultural settings.
(2) Integration of different trackers with Detectron2 detection: the study combines the detection capabilities of Detectron2 with various trackers such as Simple Online Real-time Tracking (SORT), Deep SORT, Modified Deep SORT, ByteTrack, Centroid, Centroid with Kalman filter, IOU, and our CTA.
(3) Modification of the Deep SORT tracking algorithm: the study modifies the Deep SORT tracking algorithm by combining it with a modified re-identification process, leading to improved performance in cattle tracking.
(4) Implementation of CTA: The study leverages the IOU bounding box (BB) calculation and finds the missing track-IDs for the re-identification process. Our CTA offers accurate and reliable cattle tracking results and minimizes the occurrence of identification switch cases caused by different occlusions. It can also control duplicated track-IDs and handle cases without track-ID increments for miss detection to test long-time videos. Our CTA has the remarkable ability to retain the original track-ID even in cases of track-ID switch occurrences.
(5) In addition to the above contributions, the research focuses on developing a real-time system for the automatic detection and tracking of cattle. The research addresses the challenge of accurately detecting foreground objects, particularly cattle, amidst a mixture of other objects, such as people and various background elements. Furthermore, the study tackles the complexity of track-ID switch cases in large-size calving pen environments, specifically addressing the occlusion challenges encountered when tracking cattle.

This paper is structured into five sections. Section 1 introduces the research. Section 2 discusses the research background and presents relevant works in the field. Section 3 describes the materials utilized for the analysis and explains the methods employed in this study. In Section 4, the experimental implementation results and analysis are presented in detail. Section 5 presents the discussion of this research. Finally, Section 6 presents the conclusions of this proposed research.

## 2. Research Background and Related Works

The accurate prediction of calving events is crucial for dairy farm management as it allows personnel to determine the need for assistance [9]. Timely help during calving is essential to avoid prolonged labor and potential health issues for both the mother cow and calf. Automatic and precise cattle tracking plays a significant role in seamless cow monitoring during calving, enabling dairy farmers to address challenges proactively, improve herd welfare, and optimize reproductive performance.

Cattle tracking has gained significant importance in various domains, including precision agriculture, livestock management, and animal behavior analysis [10]. Traditional methods of cattle tracking, such as manual observation or manual tagging, are time-consuming, labor-intensive, and prone to human errors. Deep learning-based object detection and tracking algorithms [11,12] have shown remarkable success in various applications, including pedestrian tracking, vehicle tracking, and object recognition [13,14,15]. However, when applied to cattle tracking, these algorithms face unique challenges. One of the major challenges is occlusion [16,17].

To address these challenges, researchers have explored different approaches to cattle tracking. Some studies have focused on improving the accuracy of object detection algorithms specifically for cow detection, leveraging deep learning models trained on cow-specific datasets [18]. Others have investigated methods to handle occlusion and track-ID switch cases, such as multi-object tracking algorithms that incorporate temporal information and appearance-based models for re-identification [19]. However, despite the progress made in cattle tracking research [20], there is still a need for more robust and efficient tracking systems that can effectively handle occlusion conditions and track-ID increment cases for miss detection [21]. The existing literature lacks comprehensive evaluations of different tracking algorithms [22] specifically tailored for cattle tracking. This research builds upon the existing body of knowledge in cattle tracking and aims to contribute to the field by addressing the challenges of occlusion conditions and track-ID increment cases for miss detection. The existing algorithms have their respective advantages and disadvantages. In Table 1, detailed information about the performance of existing algorithms is provided.

## 3. Materials and Methods

**Figure 1:**
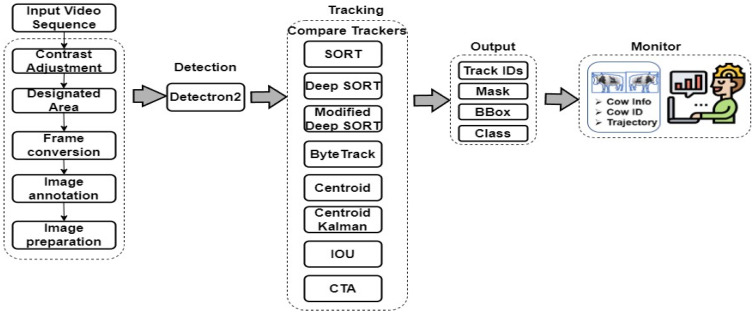
The methodology of the proposed research.

In this section, we will outline our proposed system designed to automate the monitoring of cattle detection and tracking systems, minimizing the requirement for constant human observation. Figure 1 illustrates the research methodology we propose to follow.

### 3.1. Data Preparation

**Figure 2:**
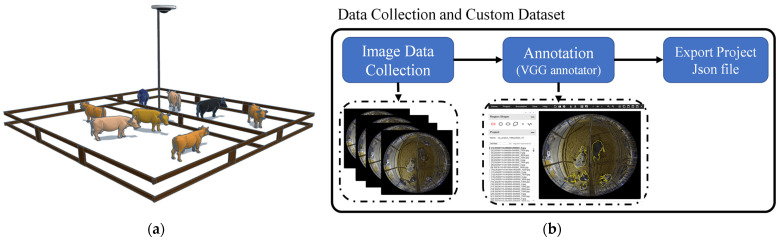
(**a**) Illustration of the camera setting; (**b**) data preparation process.

To collect the dataset at the large-scale dairy farm in Oita Prefecture, a 360-degree surveillance camera with a fisheye lens was used. The camera was set to record at a resolution of 2048 × 2048 pixels and a frame rate of 30 fps (frames per second). After collecting these images from the original video, the training and validation images were separated. VGG annotator [23] was used to annotate the cow regions. An illustration of the camera setting and the detailed process for data preparation are presented in Figure 2.

This research work was annotated using calving data images from videos with 1 frame per minute, and the dataset contains 1320 images. Among them, we used 1080 images that included 7725 cow instances for training. For validation, we utilized 240 images that involved 2375 cow instances. We used 80% for training and 20% for validation. In Table 2, detailed information about the dataset is presented.

### 3.2. Data Preprocessing

#### Contrast Adjustment and Designated Area

**Figure 3:**
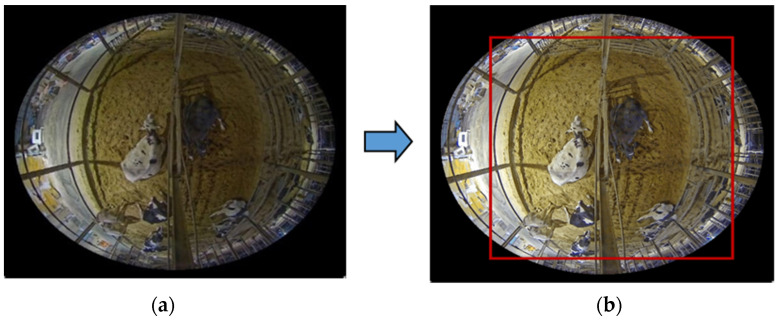
(**a**) Original video; (**b**) added contrast within the designated area.

For nighttime video testing, the lighting conditions may have been low, and contrast adjustment was still necessary to enhance the visibility of the cattle in the video frames. By applying a contrast function, the algorithms could better differentiate between the foreground (cattle) and the background, leading to more accurate and reliable tracking results. The proposed research demonstrates the process of defining a ROI using coordinates (x, y, w, and h) as (339, 261, 1330, and 1592) and creating corresponding masks. The results of this test showcase the application of ROI masks for focusing on specific regions of interest within an image. In Figure 3, there is a comparison between the original video and the video after applying contrast within the designated area.

### 3.3. Cattle Detection

The proposed system utilizes the Detectron2 framework for automatic cow detection, leveraging deep learning techniques to accurately locate and identify cows. Detectron2 is an open-source software library designed for object detection and segmentation built on top of PyTorch [24,25]. The customized Detectron2 detection model can undergo fine-tuning using transfer learning, an effective technique that leverages the pre-trained model’s weights to enhance its performance on a cattle dataset. By utilizing transfer learning, the model can learn to identify distinctive features of cattle, such as their shape, size, and color, thereby improving its accuracy in detecting cattle.

#### Noise Removal

**Figure 4:**
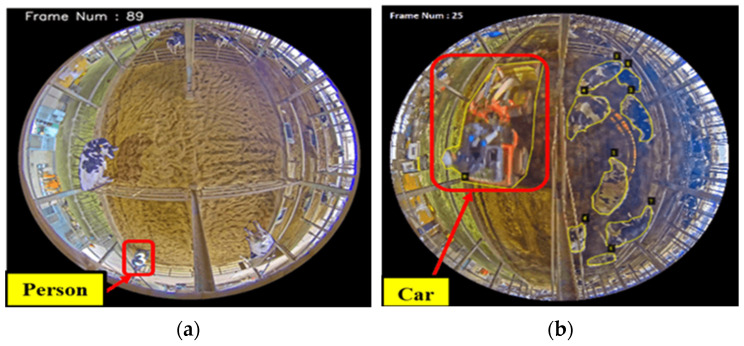
(**a**) Noise removal (person); (**b**) noise removal (car).

Our Detectron2 cow detection model also incorporated human and vehicle regions, such as those that might enter and exit the calving pen. To solve these problems, the vehicle and human regions from the detected frames were filtered out. This was achieved by calculating the area of the binary mask and comparing it to a predetermined threshold. Below this threshold, any detected object in a mask area was labeled as a noise zone and subtracted from the detection results. By excluding these areas, the approach prioritizes cow data, resulting in more precise and reliable analysis in terms of cattle detection. The noise reduction for the human and tank car zones is shown in Figure 4. Here, we define the detected cow region as the area that falls within the range of Threshold 1 (*Th*_1_ = 5000) and Threshold 2 (*Th*_2_ = 30,000).

### 3.4. Cattle Tracking

In this research, the cattle tracking process is performed on the bounding box predictions obtained from the detection stage compared with eight different deep learning algorithms: SORT, Deep SORT, Modified Deep SORT, ByteTrack, Centroid, Centroid with Kalman filter, IOU and CTA.

#### 3.4.1. SORT Algorithm

**Figure 5:**
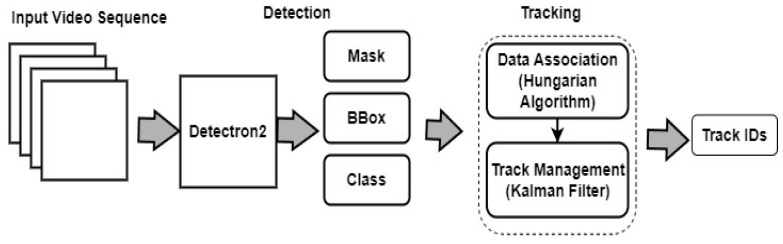
Architecture framework of the Detectron2 and the SORT algorithms.

SORT is an online tracking algorithm that is simple and computationally efficient [26]. It works by first detecting objects in each frame of a video, then assigning a unique ID to each object and tracking it across frames. SORT utilizes the Hungarian algorithm for assigning IDs to the cattle while also incorporating a Kalman filter to estimate the state of each cow over time. Figure 5 illustrates a detailed architecture framework that integrates Detectron2 and the SORT Algorithm.

#### 3.4.2. Deep SORT Algorithm

**Figure 6:**
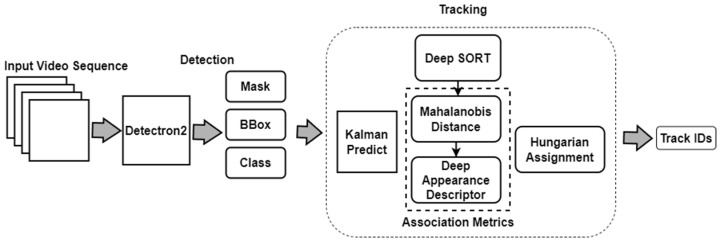
Architecture framework of the Detectron2 and Deep SORT algorithms.

Deep SORT is an extension of SORT that incorporates deep learning features to improve tracking performance [27]. In addition to the Hungarian algorithm and Kalman filter, Deep SORT uses a deep appearance feature extractor to calculate similarity scores between cattle in different frames. This helps to reduce the number of ID switches and improve overall tracking accuracy. Figure 6 illustrates a detailed architecture framework that integrates Detectron2 and the Deep SORT algorithm.

#### 3.4.3. Modified Deep SORT Algorithm

**Figure 7:**
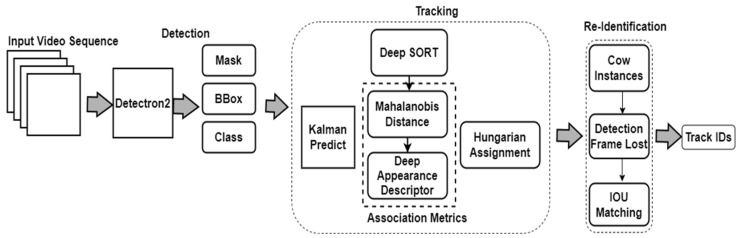
Architecture framework of Detectron2 and Modified Deep SORT algorithms.

In the Modified Deep SORT algorithm, we have made specific modifications to enhance the re-identification process. Firstly, we determined the number of cow instances by calculating the total number of bounding boxes. Subsequently, we evaluated the detection frame loss and IOU for each of these bounding boxes. If no detection occurred for a particular frame, we proceeded to check the overlap between all of the IOU bounding boxes. If there was any overlap detected, we calculated the IOU metric for each pair of bounding boxes, allowing us to identify the overlapping bounding boxes and their corresponding IDs. Specifically, if the IOU between two bounding boxes was greater than or equal to 0.9, we retained the values of these bounding boxes and their respective IDs. These modifications ensured a more effective re-identification process within the Modified Deep SORT algorithm. Figure 7 illustrates a detailed architecture framework that integrates Detectron2 and the Modified Deep SORT algorithm.

#### 3.4.4. ByteTrack Algorithm

The ByteTrack algorithm [28] utilizes the Kalman filter state vector to predict and associate tracking boxes. However, the constant velocity model assumption of the Kalman filter can result in suboptimal bounding box shapes compared to the detections obtained from the Detectron2 detector. The Kalman filter state vector estimates the aspect ratio of tracking boxes, causing inaccurate width estimation in the ByteTrack algorithm.

**Figure 8:**
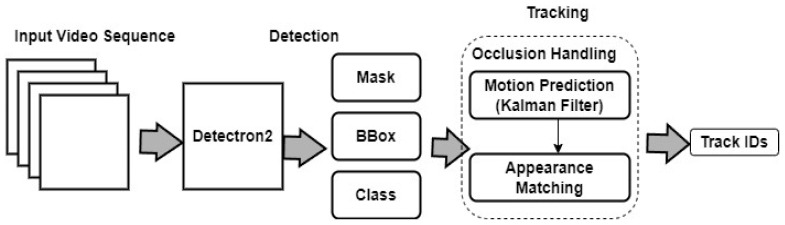
Architecture framework of the Detectron2 and ByteTrack algorithms.

To address the issues with ByteTrack, we have implemented several modifications to enhance its performance. Firstly, we have incorporated a lightweight backbone network that significantly reduces the computational overhead. This allows for faster processing and real-time tracking speeds. Additionally, we have introduced a sparse tracker that focuses on tracking a subset of the detected cattle in each frame. By carefully selecting relevant targets for tracking, we improved the overall tracking accuracy while maintaining efficiency. These modifications have proven to be highly effective in addressing the challenges posed by ByteTrack. Our revised approach achieves exceptional tracking accuracy while ensuring that the system operates at real-time speeds. Specifically, enhancements were implemented to address issues related to occlusion and tracking speed. By refining the original ByteTrack algorithm, we achieved notable improvements in testing duration compared to other state-of-the-art multi-object tracking algorithms such as SORT and Deep SORT. Additionally, our modified ByteTrack algorithm demonstrated enhanced capabilities in handling occlusion cases to a certain extent. In Figure 8, a detailed architecture framework is presented, showcasing the integration of the Detectron2 and ByteTrack algorithm.

#### 3.4.5. Centroid Tracking Algorithm

**Figure 9:**
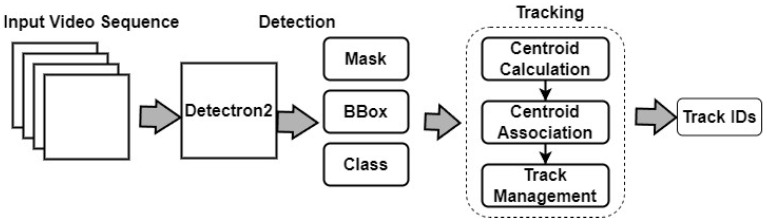
Architecture Framework of the Detectron2 and Centroid Tracking algorithms.

Centroid [29] is a simple tracking algorithm that estimates the centroid (i.e., center point) of each cow in each frame and tracks it over time. It assumes that the centroid of a cow is a stable and reliable feature that can be used to identify it across frames. Figure 9 illustrates the detailed architecture framework of the Detectron2 and Centroid Tracking algorithms.

#### 3.4.6. Centroid with Kalman Filter Algorithm

**Figure 10:**
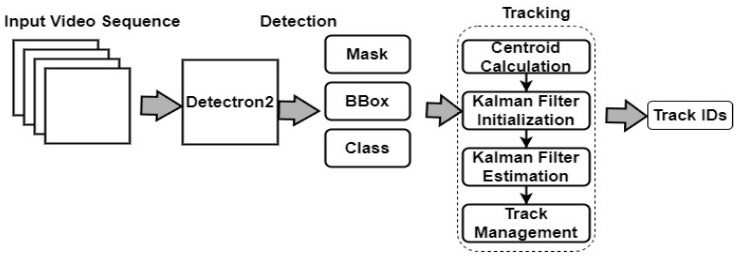
Architecture framework of the Detectron2 and Centroid with Kalman filter.

Centroid can be combined with a Kalman filter to improve tracking performance. The Kalman filter [30] estimates the state of each cow (e.g., position, velocity) based on its previous state and the current centroid measurement. Figure 10 illustrates a detailed architecture framework that integrates Detectron2 and the Centroid with Kalman filter algorithms.

#### 3.4.7. IOU Tracking

**Figure 11:**
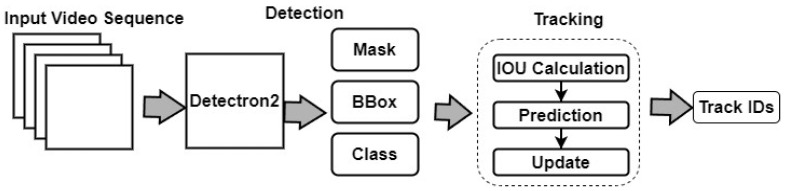
Architecture framework of Detectron2 and IOU tracking.

IOU [31] is a simple online tracking algorithm that uses the IOU metric to associate cattle in different frames. The IOU measures the overlap between two bounding boxes, and cows with high IOU values are assumed to be the same cow. IOU tracking is computationally efficient and can handle large numbers of cattle, but it may suffer from ID switches and fragmentation. However, we discovered that Detectron2, coupled with the IOU method, excels in accurately detecting and tracking individual cows, even in complex environments. It outperforms other algorithms in terms of accuracy and computational efficiency. The Detectron2 coupled with IOU is shown in Figure 11.

#### 3.4.8. Customized Tracking Algorithm (CTA)

**Figure 12:**
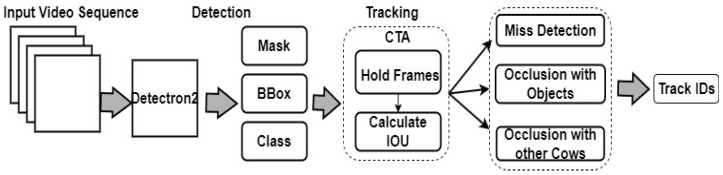
Architecture framework of the Detectron2 and CTA.

Unlike many existing algorithms, our CTA has successfully addressed three significant challenges in the field of multi-object tracking. Firstly, our CTA effectively tackles miss detection, ensuring that cattle are rarely overlooked or lost during the tracking process. Secondly, our CTA demonstrates exceptional performance in handling occlusion scenarios involving objects such as camera stands. Whether targets are partially or fully obstructed by other objects in cluttered scenes or crowded environments, CTA remains adept at maintaining precise tracking, providing reliable results even in challenging conditions. Lastly, one of the most remarkable aspects of our CTA is its ability to cope with occlusions caused by other cows. In scenarios where multiple cows are present and may obstruct each other from the camera’s view, our CTA remains resilient and ensures continuous and accurate tracking of individual cattle. The Detectron2 coupled with the CTA working structure is shown in Figure 12.

##### CTA—Miss Detection

**Figure 13:**
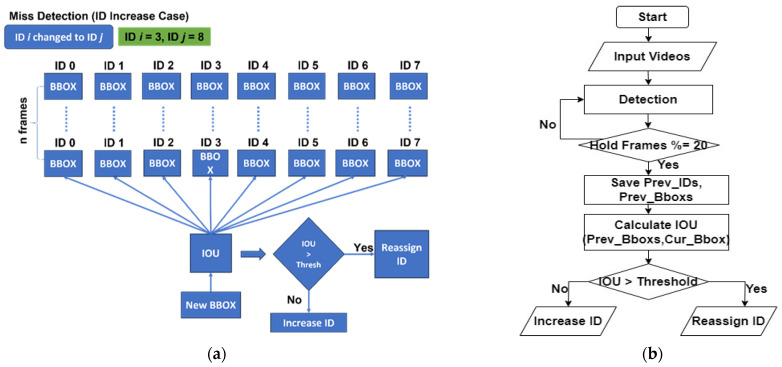
Solving process used in CTA miss detection: (**a**) logic explanation; (**b**) flow chart.

Miss detection occurs when the tracking algorithm fails to detect or temporarily loses track of a cow. To mitigate this issue, our algorithm takes a proactive approach by storing the IDs and bounding boxes of objects for the previous *n* frames. As shown in Figure 13, first, we stored all the bounding boxes and their corresponding IDs for *n* frames. We assumed *n* is 20. Then, sometimes, a detection might be missed, but later, it is re-detected. In such cases, we calculated the new bounding boxes and saved them. When a new bounding box was obtained, we compared it with the stored bounding boxes using IOU. If the IOU exceeded a specified threshold, the algorithm reassigned the stored ID to the new bounding box, maintaining consistent tracking. However, if the IOU was below the threshold, the algorithm assigned a new ID to the object, treating it as a separate entity and preventing the risk of losing track of the target. Overall, our algorithm effectively handles miss detection scenarios, ensuring accurate and reliable cattle tracking. Figure 13 depicts a detailed resolution process for handling missed detections, showcasing the implementation of the CTA.

**Figure 14:**
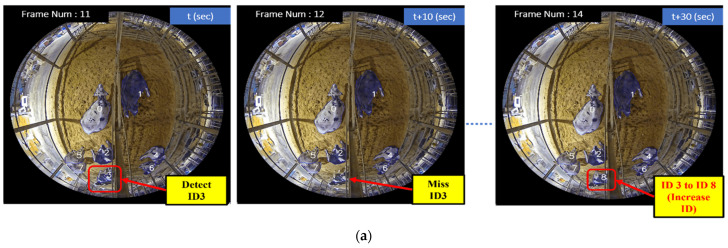

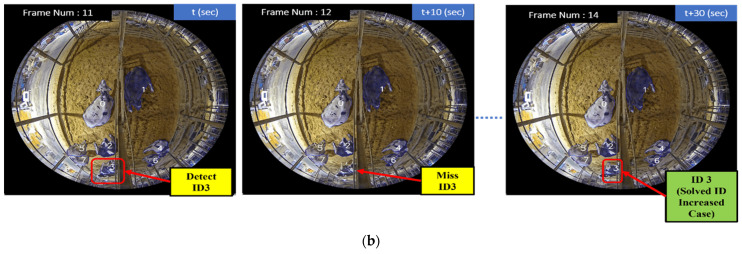
Comparison of miss detection case when using IOU and CTA: (**a**) IOU algorithm (which cannot solve the ID increment case); (**b**) CTA (which solved the ID increment case).

In Figure 14, we can see that the ID 3 cow is detected in Frame Number 11 at *t* second. Frames are calculated at a rate of ten per second. In Frame Number 12, this cow is mistakenly not detected (missed detection). However, in Frame Number 14, the same cow with ID 3 is re-detected. If we use only the IOU-based algorithm for ID assignment, there is a chance that the cow with ID 3 might be assigned a new ID (ID 8) because the IOU between the missed detection (Frame 12) and the re-detected bounding box (Frame 14) might be below the specified threshold. This could lead to ID increases. However, with our CTA, we handle this situation differently. Instead of strictly relying on the IOU-based algorithm, we take into consideration the previous history of the cow with ID 3. Since we had previously seen this cow with ID 3 in Frame 11, we recognized it despite the missed detection in Frame 12. When the same cow reappears in Frame 14, we associate it back to the original ID 3, maintaining consistent tracking. Holding the previous frames for each ID’s bounding box allows our tracking algorithm to maintain consistent tracking even when a cow reappears after being temporarily missed.

##### CTA—Occlusion with Objects

**Figure 15:**
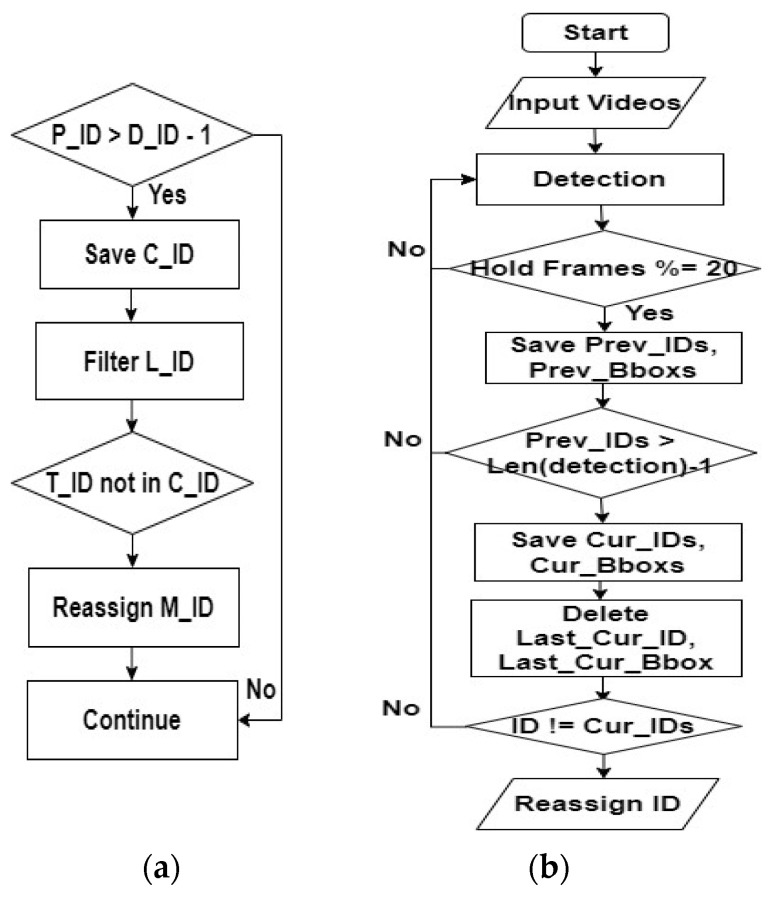
Solving process for CTA—occlusion with objects: (**a**) logic explanation; (**b**) flow chart.

Our CTA exhibits remarkable proficiency in coping with different occlusion scenarios that involve objects like camera stands. Our CTA mainly uses IOU bounding box calculations with a re-ID process. In the case of occlusion with objects, it reassigns the missing ID to the new track and deletes the incorrect track. Here, we assume that n would be 8. Figure 15 illustrates the solving process for CTA—occlusion with objects for (a) logic explanation and (b) flow chart explanation.

This is the logic explanation. If the number of previously tracked IDs (*P_ID*) is greater than the total number of detections (*D_ID*) minus one, the algorithm proceeds with the following steps. It saves the Current_IDs (*C_ID*), which represent the IDs of all currently detected objects. Next, it filters out the last tracked ID (*L_ID*) from the previous frames. After that, the algorithm checks if the track_num (*T_ID*) is not present in the *C_ID* list. If the *T_ID* is not found in the *C_ID* list, it means this ID was missing in the current frame. In such cases, our algorithm calculates the miss ID (*M_ID*), which represents the number of consecutive frames this ID has been missing. Subsequently, the algorithm reassigns a *M_ID* to the *C_ID* list. By following these steps, the algorithm ensures consistent tracking and effectively handles missing detections during cattle tracking while providing unique IDs for the missed cattle.

**Figure 16:**
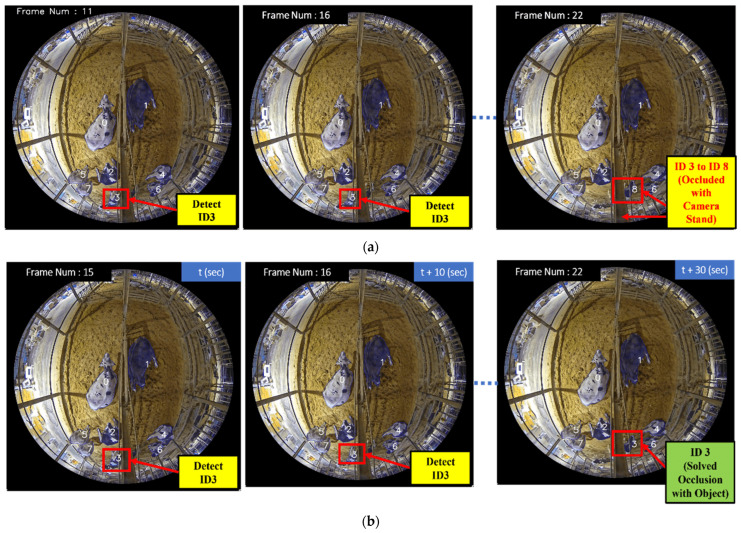
Comparison of occlusion with object case of using IOU and CTA: (**a**) IOU algorithm (which cannot solve the ID increment case); (**b**) CTA (which solved the ID increment case).

This is the flow chart explanation. Initially, the input videos are processed to detect all of the cows present. Subsequently, to maintain consistency in tracking, we implemented a strategy of holding the detection list for every 20 frames, ensuring continuity in assigning IDs and BBoxs for each cow over this duration. Within each 20-frame interval, we store the IDs and Bboxs for all of the detected cows. After 20 frames, these data are refreshed for the next set of frames. If the number of previously tracked IDs is greater than the total number of detections minus one, our algorithm proceeds with the following steps. It saves the current IDs and their respective current Bboxs in a list. Next, it filters out the last tracked ID and its corresponding Bbox from the list. After that, the algorithm checks if the ID is not present in the current ID list. If the ID is not found in the current ID list, it means this ID was missing in the current frame. In such cases, our algorithm assigns this ID to miss ID and reassigns such ID to the current list. In Figure 16, a detailed resolution process for handling occlusion with object is depicted, showcasing the implementation of the CTA.

In Figure 16, a cow with ID 3 is detected in Frame Number 15 at second t. The frames are also calculated at a rate of ten per second. However, in the subsequent Frame Number 16, this cow is partially occluded by an object, in this case, the camera stand, leading to a missed detection. Consequently, ID 3 is not detected in Frame 16. If we were to rely solely on the IOU-based algorithm for ID assignment, there would be a risk of mistakenly assigning a new ID (ID 8) to the cow when it reappears in Frame Number 22. However, in our CTA, upon detecting the cow again in Frame Number 22, the new ID 8 is deleted, and the IOU is recalculated. This allows our CTA to recognize that the re-detected cow corresponds to the cow that was missed in Frame 16, and therefore, it correctly reassigns the original ID 3 to the cow. This way, the occlusion issue is appropriately handled, and the tracking remains reliable and accurate throughout the video sequence.

##### CTA—Occlusion with Other Cows

**Figure 17:**
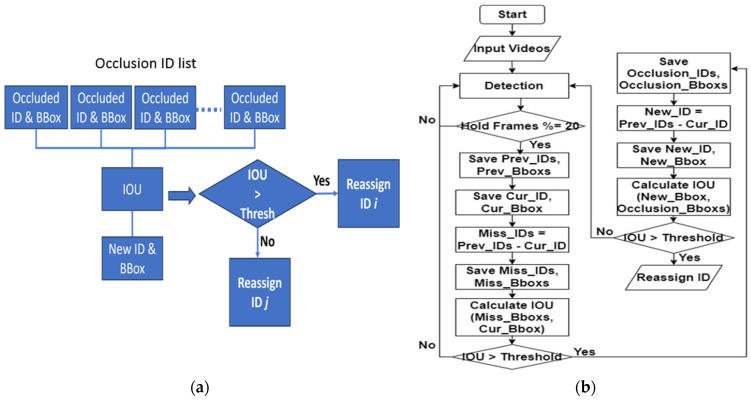
Solving process for CTA—occlusion with other cows: (**a**) logic explanation; (**b**) flow chart.

Figure 17 illustrates the solving process for CTA—occlusion with other cows for (a) the logic explanation and (b) the flow chart explanation. This is the logic explanation for Figure 17. Our CTA effectively handles occlusion scenarios with other cows. It accomplishes this by first saving dictionaries that contain IDs and bounding boxes of previous and current tracks. By comparing these tracks, the algorithm identifies missed detections and computes the IOU to detect potentially occluded cows where the bounding boxes overlap. The resultant occlusion cow IDs and bounding boxes are stored in a list for further analysis. To distinguish between occludee and occluder cows, our algorithm assigns a unique ID to the re-detected cows (occludee cows). Subsequently, our CTA calculates the IOU between the newly assigned ID and the saved occlusion IDs from the occlusion list. If the IOU exceeds the specified threshold, our algorithm reassigns the ID *i* to the cow. If the IOU is below the specified threshold, our algorithm reassigns the ID *j* to the cow. Here, IDi refers to the track_ID with the highest IOU values within the occlusion list, signifying the track with the strongest association with the occluded cows. ID *j* represents the new track_ID and its corresponding BBox that emerges during the occlusion event. This approach enhances the reliability of ID switch cases, especially in complex scenarios with occlusions, leading to improved tracking performance.

This is the flow chart explanation. Initially, the input videos are processed to detect all cows present. Subsequently, to maintain consistency in tracking, we implemented a strategy of holding the detection list every 20 frames, ensuring continuity in assigning IDs and BBoxs for each cow over this duration. Within each 20-frame interval, we stored the IDs and Bboxs for all of the detected cows. After 20 frames, these data are refreshed for the next set of frames. Firstly, it saves previous IDs and their respective previous Bboxs. After that, it saves current IDs and their respective current Bboxs. We can calculate the miss ID and its Bboxs by comparing the previous to the current. After that, we calculate the IOU for the miss Bboxs and current Bboxs. If the IOU is greater than the assigned threshold, we save the IDs and their Bboxs as an occlusion list. Then, we calculate the new ID and new Bbox by comparing the previous to the current again. We calculate the IOU once more for the new Bboxs and occlusion Bboxs. If the IOU is greater than the assigned threshold, we reassign this ID and its Bbox.

**Figure 18:**
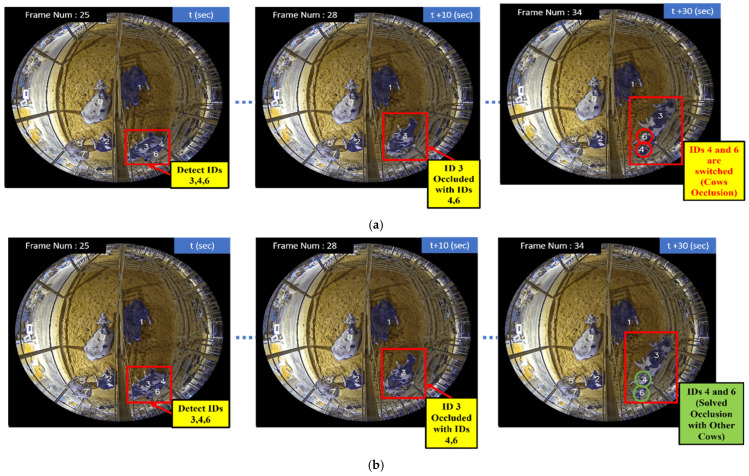
Comparison of occlusion with other cows of using IOU and CTA: (**a**) IOU algorithm (which cannot solve ID switch case); (**b**) CTA (which solved ID switch case).

In Figure 18, we observe that cow IDs 3, 4, and 6 are detected in Frame Number 25 at second t. The frames are calculated at a rate of ten per second. However, in Frame Number 28, cows with IDs 4 and 6 become occluded by cow ID 3, resulting in a missed detection for IDs 4 and 6. Due to this occlusion, the cows with IDs 4 and 6 are not detected in Frame Number 28. If we were to rely solely on the IOU algorithm for ID assignment, there could be a case of ID switching for 4 and 6 when they reappear in Frame Number 34. However, our CTA ensures that such occlusion scenarios are handled correctly. When the cows with IDs 4 and 6 are detected again in Frame Number 34, our CTA recognizes that they were previously missed due to occlusion and correctly assigns their original IDs (4 and 6).

## 4. Experimental Implementation Results and Analysis

The testing computer runs on Windows 10 Pro with a powerful 3.20 GHz 12th Gen Intel Core i9-12900K processor with 64 GB of memory. It also features a spacious 1 TB HDD and a high-performance NVIDIA GeForce RTX 3090 GPU. The system specification for execution is presented in Table 3.

To thoroughly examine the performance of the proposed system, we conducted two experiments: (1) a dairy cattle detection experiment using the Detectron2 customized cattle detection and segmentation algorithm and (2) a dairy cattle tracking experiment to compare the performance of eight deep learning tracking algorithms for tackling occlusion and miss detection.

### 4.1. Performance Analysis of Cattle Detection Results

To evaluate the detection performance, we computed three types of average precision (AP) values for both bounding box (BB) and mask predictions. The AP values were calculated at various IOU thresholds ranging from 0.5 to 0.95, with intervals of 0.05. Table 4 presents the training and validation accuracies for the detection model.

Regarding the execution time, the training process for our detection model took approximately 23 min and 18 s for 1320 images. The execution time of the training phase is influenced by the complexity of the model architecture, the size and complexity of the dataset, the hardware specifications of the computational resources, and the optimization techniques employed.

#### 4.1.1. Nighttime Detection Accuracy and Results

Table 5 presents the evaluation results for different cameras over a specific period at nighttime. One frame per second from each one-hour duration video is tested. To evaluate the detection accuracy of our system, we collected testing data over a period of five hours, encompassing both morning and evening sessions.

(1) $$\mathrm{Accuracy}=\frac{TP+TN}{TP+FP+TN+FN}$$

where TP: True Positive; FP: False Positive; TN: True Negative; and FN: False Negative.

**Figure 19:**
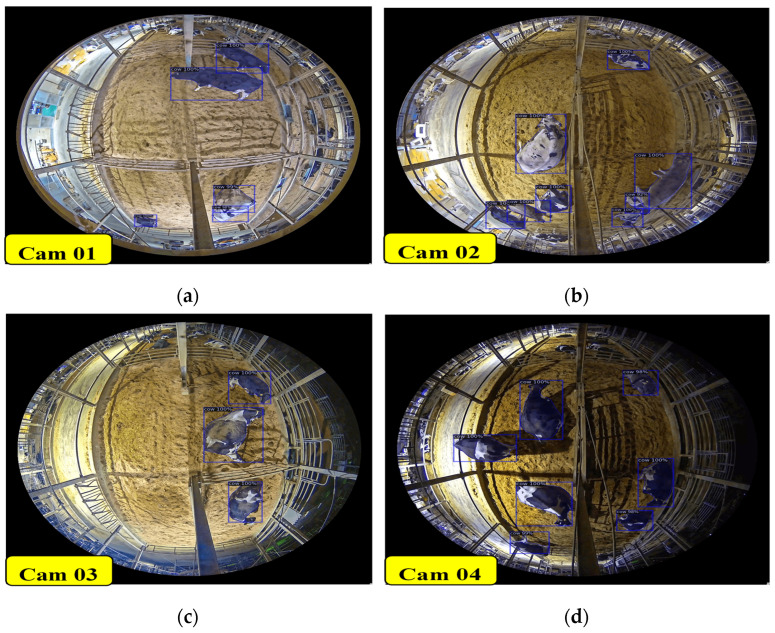
Sample detection results for all camera views at nighttime: (**a**) Cam 01; (**b**) Cam 02; (**c**) Cam 03; and (**d**) Cam 04.

Our detection model was evaluated on nighttime video sequences, as shown in Figure 19. The detection model we employed faced challenges, including instances of missing cows due to occlusion and misclassifying non-cow objects as cows. However, we have addressed these issues by incorporating the contrasting method into our model. Notably, our system achieved an impressive average detection accuracy of 99.91% across all cameras for five hours duration result, demonstrating its high performance and reliability in accurately identifying and tracking cows.

#### 4.1.2. Daytime Detection Accuracy and Results

**Figure 20:**
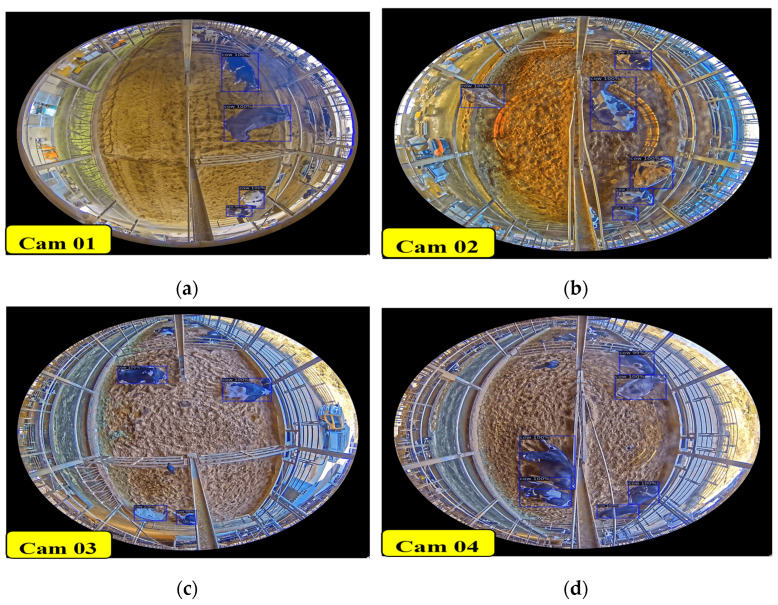
Sample detection results for all camera views during daytime: (**a**) Cam 01; (**b**) Cam 02; (**c**) Cam 03; and (**d**) Cam 04.

Table 6 presents the evaluation results for different cameras over a specific period during the daytime. Remarkably, our system achieved an impressive average detection accuracy of 99.81% across all cameras across a five-hour duration, as depicted in Figure 20.

### 4.2. Performance Analysis of Cattle Tracking Results 

To evaluate the performance of cattle tracking, we have adopted the most common evaluation metrics expressed in the MOT16 benchmark: the Multi-Object Tracking Accuracy (MOTA) metric. The MOTA calculation is defined by Equation (2).

(2) $$\mathrm{MOTA}=1-\frac{\sum_{t}\left(FN_{t}+FP_{t}+{IDS}_{t}\right)}{\sum_{t}GT_{t}}$$

where IDS: ID Switch; GT: Ground Truth; FN: Miss Tracks; and FP: False Track.

#### 4.2.1. Nighttime Tracking Accuracy and Results 

The evaluation results for automatic cattle tracking for nighttime testing videos are presented in Table 7. To calculate the accuracy, the testing data are collected from videos taken from each camera view across a 5 h duration on 10 January 2023 (00:00:00 to 05:00:00 a.m.).

**Figure 21:**
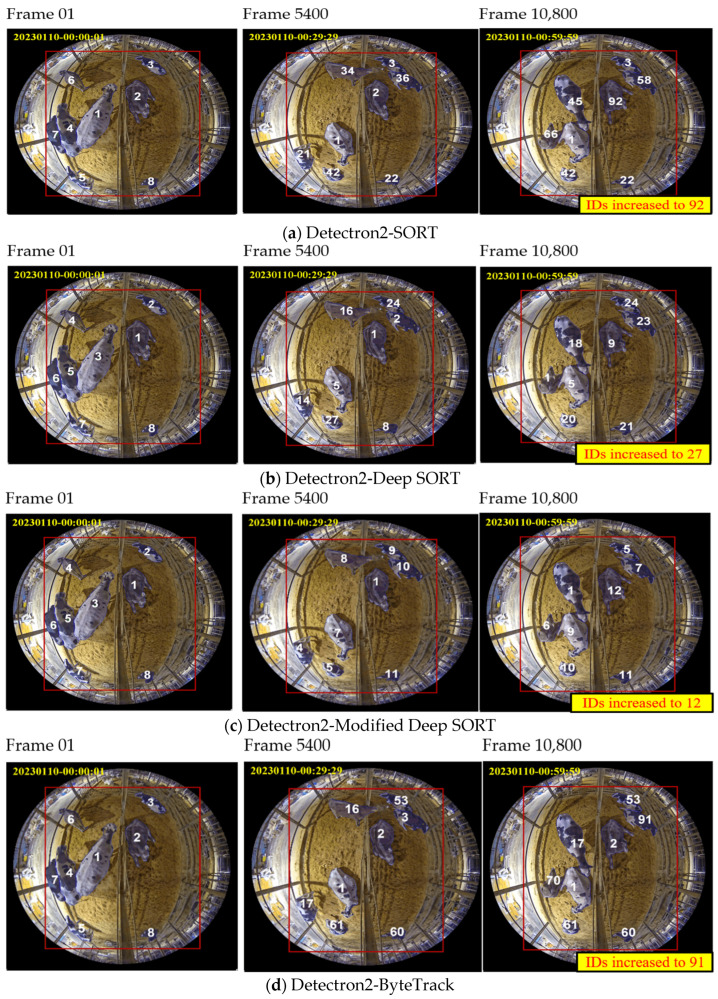

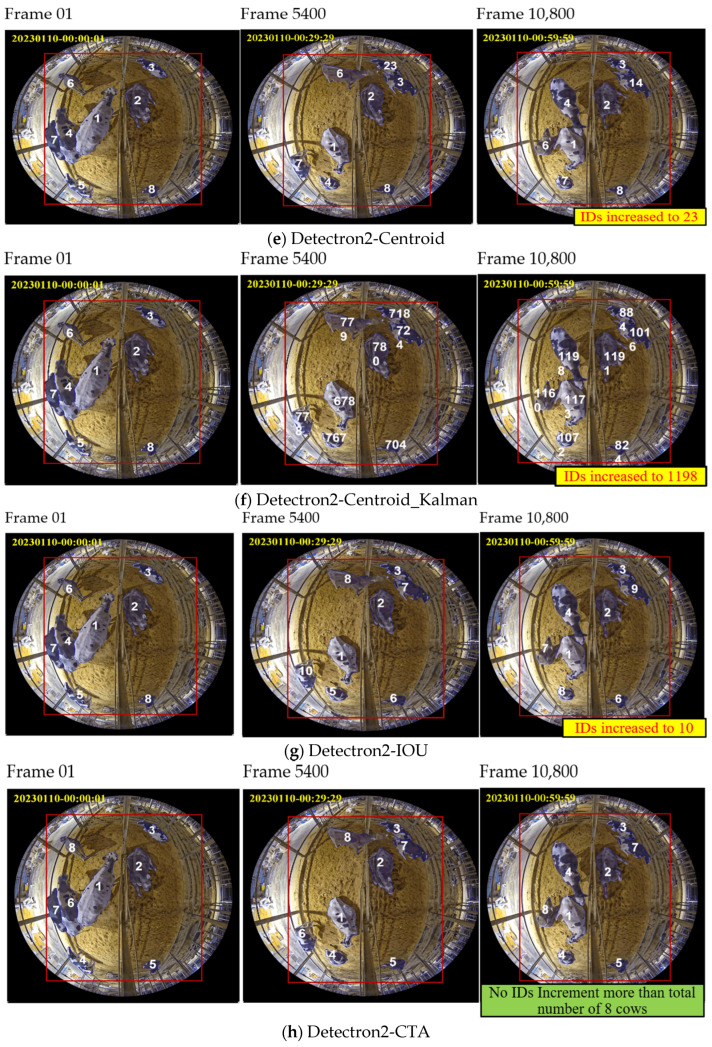
Sample tracking results using eight different trackers for Cam 03 at nighttime: (**a**) Detectron2-SORT; (**b**) Detectron2-Deep SORT; (**c**) Detectron2-Modified Deep SORT; (**d**) Detectron2-ByteTrack; (**e**) Detectron2-Centroid; (**f**) Detectron2-Centroid_Kalman; (**g**) Detectron2-IOU; (**h**) Detectron2-CTA.

Ten frames per second from each one-hour duration video were tested. GT, FP, FN, and IDS are evaluation metrics that are calculated using the number of frames. In Figure 21, a comprehensive comparison is shown, where eight different tracking methodologies are integrated with a customized detection model to analyze the cattle-crowded video captured from the view of Camera 3. It depicts a densely populated scene with multiple cattle moving and interacting with each other. The video spans a duration of 5 h and was recorded on 10 January 2023 from 00:00:00 to 05:00:00 a.m. The figure provides a detailed analysis of how cow IDs are affected, not only due to missed detections but also due to occlusions encountered during the testing duration. It showcases how each tracking methodology faces these challenges and how the cow IDs are increased or reassigned.

#### 4.2.2. Performance Analysis of Miss Detection and Occlusion Results

**Figure 22:**
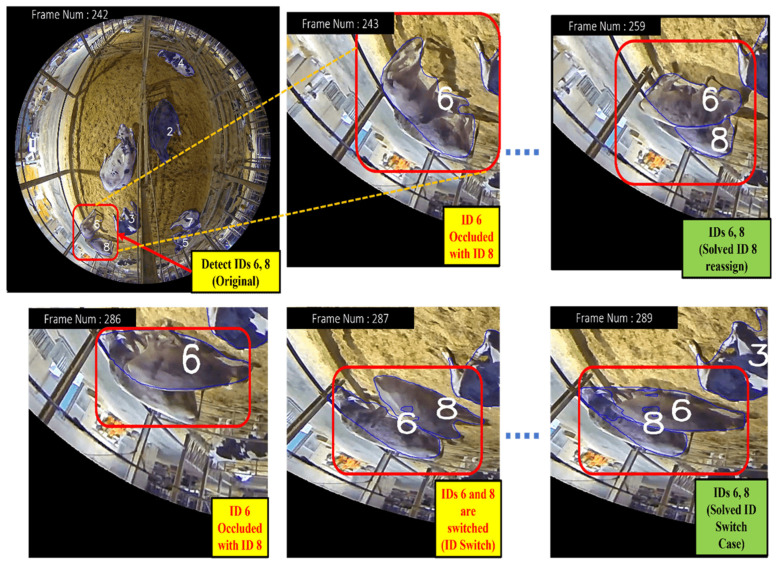
Sample performance analysis of miss detection and occlusion using CTA.

Our CTA surpasses popular tracking algorithms such as SORT, Deep SORT, Modified Deep SORT, ByteTrack, Centroid, Centroid_Kalman, and IOU, as it effectively handles miss detection and occlusion challenges. Unlike the above algorithms, our CTA successfully addresses miss detection and occlusion problems, making it a superior choice for tracking tasks. Even in the presence of full occlusion, our CTA accurately retains the original IDs, ensuring consistent and reliable tracking. Figure 22 showcases the impressive ability of our CTA to accurately retain original IDs even in challenging scenarios, which is supported by the performance analysis of miss Detection and occlusion results.

#### 4.2.3. Daytime Tracking Accuracy and Results

Table 8 presents the daytime tracking accuracy results obtained from the analysis. To calculate the accuracy, the testing data are collected from each video from every camera view with a 5 h duration on 10 January 2023 (13:00:00 to 18:00:00 p.m.). We tested ten frames per second from each one-hour duration video tested. GT, FP, FN, and IDS are evaluation metrics that are calculated using the number of frames.

**Figure 23:**
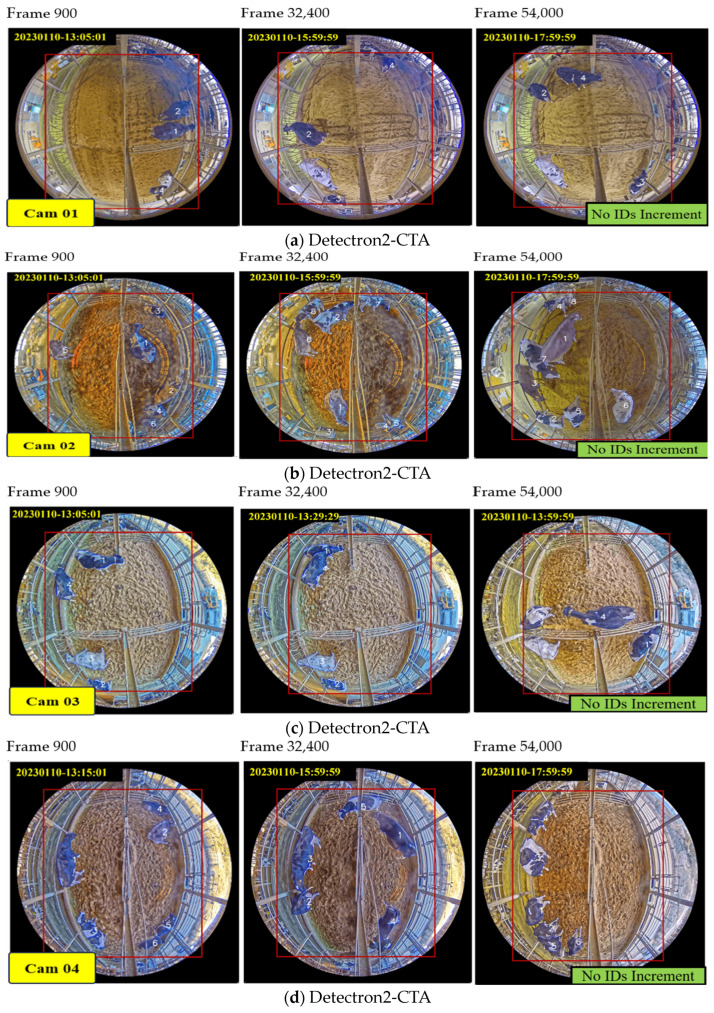
Sample tracking results using Detectron2_CTA for all cameras during daytime.

Compared to the tracking results presented in Figure 21, SORT, Deep SORT, ByteTrack, Centroid, Centroid_Kalman, and IOU algorithms display track-ID increment cases. However, the Modified Deep SORT exhibits fewer track-ID increment cases, and our CTA demonstrates no track-ID increment cases. According to our CTA results, we have avoided any track-ID duplicated cases, and we have effectively managed track-ID increment cases for instances of long-time miss detection. We can also obtain their original track-IDs even when the cows are occluded by objects (camera stands, people, and trucks). Although the tracking fails when it overlaps for long periods of time (cows occluded for longer duration), which introduces some ID switching for occludee cows, we can obtain the correct ID with the occluder cow. The incorporation of Detectron2 and our CTA leads to the best accuracy at nighttime from all camera views. Figure 23 shows the testing results from each camera view video across a 5 h duration on 10 January 2023 (13:00:00 to 18:00:00 p.m.).

In contrast with other algorithms, our proposed CTA demonstrates improved tracking abilities, even in cases of occlusion. These algorithms cannot maintain accurate tracking performance, particularly for cows with higher degrees of occlusion. Our Detectron2_CTA consistently achieves values for all camera views. These results indicate that our algorithm can continuously track targets in a variety of environments without losing track of the targets, ensuring stable tracking performance for extended durations under both nighttime and daytime conditions. Overall, our proposed method significantly improves tracking accuracy and stability compared to the other tracking methods, providing precise management support in terms of dairy farming.

#### 4.2.4. Performance Analysis of Testing Duration

Table 9 presents the calculation time for 5 h videos captured during both nighttime (00:00:00 to 05:00:00) and daytime (13:00:00 to 18:00:00) periods. The table provides valuable insights into the time taken by various methods to process the videos and perform cattle detection and tracking tasks. By presenting the testing duration for each method, this table enables a comparative analysis of the computational efficiency and performance of the tracking algorithms under different lighting conditions. This comprehensive analysis aids in identifying the most suitable tracking approach for real-world cattle monitoring applications, considering both accuracy and speed in diverse lighting environments. 

Notably, our Detectron2_CTA method emerges as the fastest and most efficient tracker when compared with other state-of-the-art tracking algorithms. Our Detectron2_CTA method excels in meeting the processing time requirements of our application. In extensive testing on 5 h videos, our Detectron2_CTA method consistently completes processing within the specified timeframe, typically taking just over 4 h but always less than 5 h. This level of efficiency sets our approach apart from the seven other algorithms we benchmarked, all of which failed to meet the application requirement due to durations exceeding 5 h for the same video length tested in Table 7. Our Detectron2_CTA method reliably delivers results within the allotted timeframe, demonstrating its suitability for processing extended video data, regardless of environmental conditions such as daytime or nighttime. Its remarkable speed allows it to run seamlessly in real-time cattle tracking systems, providing instantaneous and accurate results. The superiority of our CTA is evident in its ability to handle complex tracking scenarios, including occlusion and missed detections, with exceptional precision and reliability. The real-time capabilities of our CTA offer invaluable benefits for dairy farm management, ensuring continuous and reliable monitoring of cattle behavior. With its swift processing and accurate tracking, farmers can make informed decisions promptly, leading to improved operational efficiency and enhanced productivity. Additionally, our system’s adaptability to various lighting conditions further solidifies its suitability for practical deployment in real-world settings.

## 5. Discussion

Our proposed modified Detectron2 detection algorithm addresses the challenges of accurate and robust cattle detection by combining contrasting methods. Leveraging the strengths of different contrast methods, we can improve the overall detection performance and overcome specific challenges in cattle detection. This approach allows us to effectively handle variations in lighting conditions, complex backgrounds, occlusions, and variations in cattle appearance. Our CTA proves to be a powerful solution for overcoming occlusion challenges. By effectively managing track-IDs and handling ID increment cases for miss detection, it ensures accurate and reliable tracking results, even when cattle are partially or fully occluded by other objects. The algorithm’s ability to preserve low-score detection boxes further enhances its robustness, resilience to object deformations and precise bounding box estimation. This tailored approach not only improves tracking accuracy but also offers real-time performance, making it a valuable tool in addressing complex tracking scenarios involving occlusion. The experimental results demonstrate the outstanding performance of our proposed method on the dairy cattle detection dataset. The accuracy achieved is remarkably high, with 99.91% accuracy in nighttime conditions and 99.81% in daytime. Additionally, our Detectron2 combined with the CTA method proves highly effective for tracking, with exceptional accuracy exceeding 99% in both nighttime and daytime scenarios.

The performance analysis of testing duration demonstrates the superior speed and efficiency of our system compared to other tracking methods. Our system consistently outperforms other approaches, completing the testing tasks in significantly less time. This exceptional performance in processing data and delivering results showcases the efficiency and effectiveness of our system, making it a top-performing solution for real-world applications. By delivering accurate and precise tracking results in real time, our CTA has become an invaluable tool for livestock management, offering farmers and agricultural professionals critical insights into cattle behavior and health. Its ability to swiftly process data and provide up-to-date information empowers users to make informed decisions promptly, leading to improved herd management and optimized farming practices. The combination of high accuracy, real-time functionality, and superior speed makes our CTA a game-changer in the field of cattle monitoring and management. Its seamless performance sets a new standard for tracking algorithms, making it an indispensable asset for modern agricultural practices, ensuring healthier and more efficient dairy farm operations.

## 6. Conclusions

Our versatile system is valuable and precise cattle tracking, providing real-time tracking capabilities and accurate calving time predictions for dairy farm management. Our algorithm has demonstrated exceptional performance, surpassing 99% accuracy rates for both detection and tracking across all cameras, particularly excelling in occlusion problem-handling compared to other tracking algorithms. The focus is on developing a real-time cattle monitoring and management system that utilizes cattle trajectory tracking to offer farmers real-time guidance. The system’s implementation is crucial for accurate calving time prediction, and it excels in overcoming challenges related to miss detection and occlusion, demonstrated through extensive testing in various conditions. This comprehensive monitoring of cattle behavior enhances dairy farm management and operational efficiency, ensuring reliable and continuous cattle monitoring and ID preservation for successful tracking.

## Figures and Tables

**Table 1:** The performance of existing algorithms is shown.

No.	Existing Algorithms	Advantages	Drawbacks
1.	YOLO_SORT	Efficient tracking of multiple objects.	Susceptibility to identity switches in crowded scenes.
2.	CenterNet_ DeepSORT	Improved tracking accuracy through deep learning.	May struggle with occlusions in crowded scenes.
3.	YOLOv4_DeepSORT	Enhanced tracking accuracy with Kalman filter integration.	Increased computational complexity due to filter usage and struggle with occlusions.
4.	Probability Gradient Pattern (PGP)	Offers robustness to noise and varying illumination.	Limited effectiveness in scenes with complex backgrounds and extensive occlusions.
5.	LIBS_GMM	Effective in changing lighting conditions	Limited capability to handle complex background scenes with extensive occlusions.

**Table 2:** Dataset information.

Dataset	Date	#Frames	#Instances
Training	2021: October~November 2022: March~April, June, September~November 2023: January~February	1080	7725
Validation	2021: December, 2022: January, 2023: January	240	2375

**Table 3:** System specifications for execution.

System Component	Specification
Operating System	Windows 10 Pro
Processor	3.20 GHz 12th Gen Intel Core i9-12900K
Memory	64 GB
Storage	1 TB HDD
Graphics Processing Unit (GPU)	NVIDIA GeForce RTX 3090

**Table 4:** Training and validation detection accuracy for customized detection model.

Dataset	BBox (%)			Mask (%)		
	AP	AP 50	AP 75	AP	AP 50	AP 75
Training	92.53	98.17	95.99	90.23	97.87	95.81
Validation	91.12	97.67	95.67	89.56	97.17	95.13

**Table 5:** Detection accuracy for nighttime 5 h duration (00:00:00~05:00:00).

Cam.	Date	Period	#Frames	TP	FP	TN	FN	Accuracy (%)
01	10 January 2023	00:00:00~05:00:00	18,000	17,990	0	0	10	99.94
02				17,980	0	0	20	99.89
03				17,978	0	0	22	99.88
04				17,988	0	0	12	99.93
Average Accuracy			72,000	71,936	0	0	64	99.91

**Table 6:** Detection accuracy for daytime 5 h duration (13:00:00~18:00:00).

Cam No.	Date	Period	#Frames	TP	FP	TN	FN	Accuracy (%)
01	10 January 2023	13:00:00~18:00:00	18,000	17,990	0	0	10	99.94
02				17,980	0	0	20	99.89
03				17,978	0	0	22	99.88
04				17,988	0	0	12	99.93
Average Accuracy			72,000	71,861	0	0	139	99.81

**Table 7:** Tracking accuracy for nighttime 5 h duration (00:00:00~05:00:00).

Cam.	Methods	#Cows	GT	FP	FN	IDS	MOTA(%)
01	Detectron2_SORT	4	213,915	0	10	10,314	95.17
	Detectron2_DeepSORT			0	10	12,314	94.24
	Detectron2_Modified_DeepSORT			0	10	1610	99.24
	Detectron2_ByteTrack			0	10	3348	98.43
	Detectron2_Centroid			0	10	1401	99.34
	Detectron2_Centroid_Kalman			0	10	11,314	94.71
	Detectron2_IOU			0	10	1312	99.38
	Detectron2_CTA			0	10	3	99.99
02	Detectron2_SORT	8	354,100	0	20	11,472	96.75
	Detectron2_DeepSORT			0	20	12,413	96.49
	Detectron2_Modified_DeepSORT			0	20	4031	98.86
	Detectron2_ByteTrack			0	20	11,872	96.64
	Detectron2_Centroid			0	20	3431	99.03
	Detectron2_Centroid_Kalman			0	20	12,292	96.52
	Detectron2_IOU			0	20	2796	99.20
	Detectron2_CTA			0	20	43	99.98
03	Detectron2_SORT	8	354,100	0	22	11,517	96.74
	Detectron2_DeepSORT			0	22	12,933	96.34
	Detectron2_Modified_DeepSORT			0	22	3931	98.88
	Detectron2_ByteTrack			0	22	11,341	96.79
	Detectron2_Centroid			0	22	3231	99.08
	Detectron2_Centroid_Kalman			0	22	12,192	96.55
	Detectron2_IOU			0	22	2894	99.18
	Detectron2_CTA			0	22	48	99.98
04	Detectron2_SORT	7	323,870	0	12	10,417	96.78
	Detectron2_DeepSORT			0	12	12,243	96.22
	Detectron2_Modified_DeepSORT			0	12	1741	99.46
	Detectron2_ByteTrack			0	12	3901	98.79
	Detectron2_Centroid			0	12	1641	99.49
	Detectron2_Centroid_Kalman			0	12	10,992	96.60
	Detectron2_IOU			0	12	1413	99.56
	Detectron2_CTA			0	12	37	99.98

**Table 8:** Tracking accuracy for daytime 5 h duration (13:00:00~18:00:00).

Cam.	Methods	#Cows	GT	FP	FN	IDS	MOTA(%)
01	Detectron2_SORT	4	213,915	0	66	10,214	95.19
	Detectron2_DeepSORT			0	66	12,214	94.26
	Detectron2_Modified_DeepSORT			0	66	1510	99.26
	Detectron2_ByteTrack			0	66	3248	98.45
	Detectron2_Centroid			0	66	1301	99.36
	Detectron2_Centroid_Kalman			0	66	11,214	94.73
	Detectron2_IOU			0	66	1212	99.40
	Detectron2_CTA			0	66	4	99.97
02	Detectron2_SORT	8	354,100	0	23	11,317	96.80
	Detectron2_DeepSORT			0	23	12,213	96.54
	Detectron2_Modified_DeepSORT			0	23	3431	99.02
	Detectron2_ByteTrack			0	23	10,872	96.92
	Detectron2_Centroid			0	23	2831	99.19
	Detectron2_Centroid_Kalman			0	23	11,892	96.64
	Detectron2_IOU			0	23	2776	99.21
	Detectron2_CTA			0	23	51	99.98
03	Detectron2_SORT	8	354,100	0	32	11,417	96.77
	Detectron2_DeepSORT			0	32	12,813	96.37
	Detectron2_Modified_DeepSORT			0	32	3831	98.91
	Detectron2_ByteTrack			0	32	11,201	96.83
	Detectron2_Centroid			0	32	3131	99.11
	Detectron2_Centroid_Kalman			0	32	12,092	96.58
	Detectron2_IOU			0	32	2784	99.20
	Detectron2_CTA			0	32	34	99.98
04	Detectron2_SORT	7	323,870	0	18	10,317	96.81
	Detectron2_DeepSORT			0	18	12,113	96.25
	Detectron2_Modified_DeepSORT			0	18	1631	99.50
	Detectron2_ByteTrack			0	18	3801	98.82
	Detectron2_Centroid			0	18	1531	99.52
	Detectron2_Centroid_Kalman			0	18	10,892	96.63
	Detectron2_IOU			0	18	1313	99.59
	Detectron2_CTA			0	18	4	99.99

**Table 9:** Calculation time for 5 h videos (nighttime and daytime).

No.	Methods	Calculation Time	
		Nighttime	Daytime
1.	Detectron2_SORT	6 h 42 min	6 h 49 min
2.	Detectron2_DeepSORT	6 h 40 min	6 h 55 min
3.	Detectron2_Modified_DeepSORT	5 h 39 min	5 h 56 min
4.	Detectron2_ByteTrack	5 h 39 min	5 h 42 min
5.	Detectron2_Centroid	5 h 24 min	5 h 46 min
6.	Detectron2_Centroid_Kalman	6 h 17 min	6 h 31 min
7.	Detectron2_IOU	5 h 11 min	5 h 19 min
8.	Detectron2_CTA	4 h 45 min	4 h 51 min

## Data Availability

The datasets showcased in this study can be obtained upon request from the corresponding author.
